# Potential Role of GGBS and ACBFS Blast Furnace Slag at 90 Days for Application in Rigid Concrete Pavements

**DOI:** 10.3390/ma16175902

**Published:** 2023-08-29

**Authors:** Liliana Maria Nicula, Daniela Lucia Manea, Dorina Simedru, Oana Cadar, Mihai Liviu Dragomir, Ioan Ardelean, Ofelia Corbu

**Affiliations:** 1 Faculty of Civil Engineering, Technical University of Cluj-Napoca, 28, Memorandumului, 400114 Cluj-Napoca, Romania; daniela.manea@ccm.utcluj.ro (D.L.M.); mihai.dragomir@cfdp.utcluj.ro (M.L.D.); 2 Faculty of Construction, Cadastre and Architecture, University of Oradea, 4, B.S. Delavrancea Street, 410058 Oradea, Romania; 3 Research Institute for Analytical Instrumentation Subsidiary, National Institute for Research and Development for Optoelectronics INOE 2000, 67 Donath Street, 400293 Cluj-Napoca, Romania; dorina.simedru@icia.ro (D.S.); oana.cadar@icia.ro (O.C.); 4 Department of Physics and Chemistry, Technical University of Cluj-Napoca, 28, Memorandum Street, 400114 Cluj-Napoca, Romania; ioan.ardelean@phys.utcluj.ro; 5 Research Institute for Construction Equipment and Technology, ICECON S.A. Bucharest, 266, Pantelimon Road, 2nd District, CP 3-33, 021652 Bucharest, Romania

**Keywords:** environmentally friendly road concrete, granulated and ground blast furnace slag (GGBS), air-cooled blast furnace slag (ACBFS) aggregate, durability, hydration activity index (HAI)

## Abstract

Incorporating blast furnace slag into the composition of paving concrete can be one of the cost-effective ways to completely eliminate by-products from the pig iron production process (approximately 70% granulated slag and 30% air-cooled slag). The possibility to reintroduce blast furnace slag back into the life cycle will provide significant support to current environmental concerns and the clearance of tailings landfills. Especially in recent years, granulated and ground blast furnace slag (GGBS) as a substitute for cement and air-cooled blast furnace slag (ACBFS) aggregates as a substitute for natural aggregates in the composition of concretes have been studied by many researchers. But concrete compositions with large amounts of incorporated blast furnace slag affect the mechanical and durability properties through the interaction between the slag, cement and water depending on the curing times. This study focuses on identifying the optimal proportions of GGBS as a supplementary cementitious material (SCM) and ACBFS aggregates as a substitute to natural sand such that the performance at 90 days of curing the concrete is similar to that of the control concrete. In addition, to minimize the costs associated with grinding GGBS, the hydration activity index (HAI) of the GGBS, the surface morphology, and the mineral components were analyzed via X-ray diffraction, scanning electron microscopy (SEM), energy dispersive spectrometry (EDX), and nuclear magnetic resonance relaxometry (NMR). The flexural strength, the basic mechanical property of road concretes, increased from 28 to 90 days by 20.72% and 20.26% for the slag concrete but by 18.58% for the reference concrete. The composite with 15% GGBS and 25% ACBFS achieved results similar to the reference concrete at 90 days; therefore, they are considered optimal percentages to replace cement and natural sand in ecological pavement concretes. The HAI of the slag powder with a specific surface area equivalent to that of Portland cement fell into strength class 80 at the age of 28 days, but at the age of 90 days, the strength class was 100. The results of this research present three important benefits: the first is the protection of the environment through the recycling of two steel industry wastes that complies with European circular economy regulations, and the second is linked to the consequent savings in the disposal costs associated with wastefully occupied warehouses and the savings in slag grinding.

## 1. Introduction

Portland cement production requires a significant energy input that contributes to releasing a large amount of carbon dioxide into the atmosphere, about 7% of total global greenhouse gas emissions [1,2]. The consumption of raw materials adds 1/3 to air pollution (1 ton of cement is obtained from about 2.8 tons of limestone/shale), a process of depletion of natural resources. In addition, each tone of cement produced consumes 110–120 kWh of energy [2,3,4]. The world production of Portland cement (OPC) was 4.2 billion tons in 2021 and is expected to increase to 4.4 billion tons by the end of 2022 [5,6]. The European Union presented the circular economy as a viable alternative to the production model with an excessive and sometimes unnecessary consumption of natural resources that prevails worldwide, especially in developed countries [7,8]. The transition to regenerative systems, designed to conserve the value of resources (materials, water, land, and energy) and products, will reduce the use of raw materials and energy while reducing waste generation and negative impacts on the environment, climate, and human health [8,9].

Therefore, efficient use of renewable and non-renewable raw materials is essential for economic development [10]. Multiple wastes (fly ash, blast furnace slag, construction and demolition waste, municipal solid waste incineration ash, metallurgical and mining waste, etc.) [11,12,13,14,15,16,17] have been studied as raw materials for cement-based materials. Of these, blast furnace slag represents a sustainable source to replace conventional materials in concrete compositions. The slag can have a crystalline or glassy, amorphous structure in its solid state. GGBS is obtained by cooling liquid slag under a powerful jet of cold water, which vitrifies during the granulation process. Air-cooled blast furnace slag (ACBFS) is obtained by slowly cooling liquid slag. The resulting material crystallizes and retains a small portion in the glassy state [8]. The properties of ACBFS, its stability, and the leaching of heavy metals meet all the requirements as a building material [18] and are often used both as fine and coarse aggregates in concrete compositions [19,20,21]. ACBFS particles have a very rough texture due to their vesicular structure, but the pores present in ACBFS particles are not interconnected. However, the water absorption is higher, reaching up to 8% compared to natural aggregates where the upper limit is 4% [20]. A beneficial effect for concrete curing can be achieved in composite materials by carefully designing the mixing ratios to account for the water absorbed by the lightweight fine aggregates (LWAs) before the cement sets. These beneficial aspects include increased hydration, leading to a higher compressive strength, reduced water absorption, and reduced electrical conductivity (permeability). These advantages also include lower autogenous shrinkage and a lower tendency to early cracking [22,23].

With the development of high-performance green concrete, GGBS has become an important auxiliary material in cement concrete, also known as the “sixth ingredient” of concrete [24]. The widespread use of GGBS as a cement substitute in concrete composition is currently leading to technological, economic, and environmental benefits. GGBS is widely recommended, as it improves mechanical properties, corrosion resistance, and chloride ion penetration. In addition, well-proportioned and well-preserved slag concretes have been shown to prevent harmful alkali–silica interactions during the curing period and increase sulfate resistance. The pozzolanic action of slag used as a micro aggregate and filler (fine mass integrated into the cement matrix) resulted in a higher performance in the concrete with GGBS. At 56 days, its strength development matched conventional cement concrete’s [25]. The incorporation of fly ash (FA) or GGBS into concrete resulted in 91-day compressive strengths similar to conventional concrete. In addition, the pore structure of the concrete was changed, and the volume of capillaries larger than 0.05 μm was reduced [26]. Moreover, GGBS plays an important role in filling voids between raw materials and reducing porosity so as to increase the compactness of concrete [27,28]. Adding GGBS decreases the permeability and increases the chemical stability of self-consolidating concrete (SCC) due to the reaction of blast furnace slag with excess soluble calcium hydroxide [29,30], while ACBFS aggregates affect the flexural strength, but they improve other properties, such as the compressive strength and drying shrinkage [31].

The original contribution of this study lies in the detailed analysis of some concretes containing GGBS as supplementary cementitious material (SCM) together with this type of ACBFS as a fine aggregate in order to obtain an ecological and economical material without sacrificing mechanical performances or the durability of the obtained concrete. This work complements the current research, which is mainly concerned with the separate incorporation of GGBS or ACBFS into concrete compositions at 28 days of curing age.

The results show the optimal percentages of substituting conventional materials with blast furnace slag whose performances at 90 days of age are similar to those of the control concrete. The HAI of the GGBS was evaluated, and the slag mortars’ microstructure was analyzed via X-ray diffraction, energy dispersive electron microscopy (SEM), and nuclear magnetic resonance (NMR) relaxometry [32,33]. In addition, the influence of the specific surface area of the slag powder on the HAI and the degree of crystallinity compared to the standard mortar was analyzed up to an age of 90 days, similar to the study [34].

This research has significant implications for developing slag concrete for road paving. The extension of the reference tests from 28 to 90 days confirms the performance improvement, encouraging its use in the composition of road concretes with slag.

## 2. Materials and Methods

### 2.1. Materials

The Portland cement -CEM I 42.5R supplied by Romcim Targu-Jiu, CRH Romania Company is defined according to SR EN 197-1 [35], and the granulated blast furnace slag sourced from Combinatul Siderurgic in Galati is defined according to SR EN 15167-1 [36]. GGBS has a vitreous mass content of 95%, and the oxide composition is made up as in Table 1. A greater than 70% concentration of silicon, calcium, aluminum, magnesium, and iron oxides in GGBS indicates that it is a suitable pozzolanic material for use as a cement replacement according to ASTM D6868 [37,38]. A calcium-oxide-to-silicon ratio above one indicates effective hydraulic activity for GGBS [37,39].

River sand and ACBFS in size (0/4) mm were used as fine aggregates according to SR EN 12620 [40]. The oxide composition of the ACBFS aggregates in Table 1 is similar to that of GGBS, the difference being in the predominance of the crystalline phase. The total sulfur content was determined to be less than 1%, which is below the limit of 2% according to SR EN 12620, and the determined iron oxide content (0.58%) is also less than the 3% value according to IS: 383 [41]. The two components are limited to eliminating the expansive reaction and disintegration of the ACBFS slag particles. The water absorption coefficient WA_24_ determined according to SR EN 1097-6 [42] on found aggregates in the saturated dry surface (SSD) state was 2.02% for natural sand and more than 2.56% for ACBFS [43]. The quality assurance of the coarse aggregate from gravel (4/8) mm and for crushed quarry stone (8/16) mm was controlled according to SR EN 12620 [40] and according to SR 667 [44] for crushed quarry stone (8/25) mm.

#### 2.1.1. Mortar Mixes

**Figure 1:**
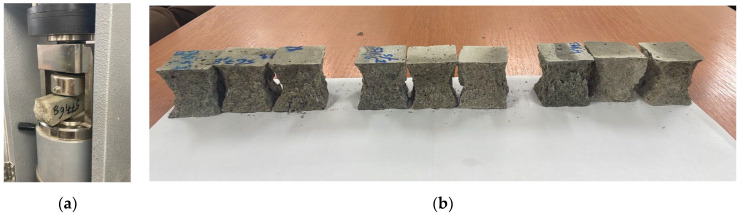
(**a**) Compressive strength test on mortars; (**b**) mortar samples after testing.

The amounts of material for the mortar production are listed in Table 2. Three mortar mixes were prepared, a standard M I with 100% Portland cement and two with 50% GGBS and with different specific surface areas, 360 m^2^/kg and 330 m^2^/kg, called M II/360 and M III/330. For the specific surface area, the air permeability method (Blaine) was used on cement particles with GGBS below 63 µm in comparison to a reference material sample according to SR EN 196-6 [45]. The 40 × 40 × 160 mm prismatic samples were preserved and tested for up to 90 days according to SR EN 196-1 [46], as seen in photo (Figure 1a,b).

#### 2.1.2. Concrete Mixes

The material proportions of the poured concrete in this experiment are listed in Table 3. Three road concrete mixes were prepared, one reference and two containing 15% GGBS with a specific surface area equal to that of cement and 25% or 50% ACBFS aggregate crushed to size (0/4) mm. To achieve the desired workability, superplasticizer additive SP was used instead of adding excess water [47]. Superplasticizer additive MasterGlenium SKY 527 and air-entraining additive Master Air 9060, Master Builders Solutions Romania group, with characteristics in accordance with SR EN 934-2 [48] and potable water in accordance with SR EN 1008 [49] were used. In the concrete manufacturing process, the aggregates were saturated with the SSD dry surface to prevent reduced workability, rapid stiffening, cement hydration delay, and the occurrence of concrete shrinkage cracks [50].

The design parameters for the pavement concrete were set for class BcR 5.0, which corresponds to very heavy traffic, commissioning in the system with slip form according to the requirements specified in NE 014 [51] and SR EN 206 [52]. The flexural strength (fct, fl) was 5.5 MPa, the w/b ratio was adjusted to 0.41, the binder dosage was 370 kg/m^3^, and the occluded air content was in the range (5.0 ÷ 6.5)%. The results of the fresh concrete were within the specified limit values, and the compressive strength (fc) already reached the minimum value of 50 MPa at the age of 7 days [43], see Table 4.

### 2.2. Methods

#### 2.2.1. The Influence of HAI of GGBS on the Composition of Mortars

The hydration activity index (HAI) of GGBS was calculated with Equation (1) as the ratio between the compressive strength of 50:50 slag cement mortar (fc_GGBS_) and the compressive strength of cement mortar (fc_C_).

HAI = (fc_GGBS_/fc_C_) × 100, (%), (1)

The strength class for GGBS according to ASTM C989 and the HAI according to SR EN 15167:1 have the values given in Table 5.

#### 2.2.2. Characterization of the Microstructure of Standard Mortar and Mortars with GGBS

X-ray diffraction (XRD) was performed for phase composition analysis and scanning electron microscopy coupled with energy dispersive spectroscopy (SEM) (EDX) was performed for microstructure analysis. Fragments from the compression test on standard mortar (M I) and mortar with 50% GBS (M II/360, M III/330) up to the age of 90 days were examined.

##### X-ray Diffraction on Mortars

XRD patterns were performed on powder samples using a D8 Advance diffractometer (Bruker, Karlsruhe, Germany) with CuK α1 anode (λ = 1.5418 Å), operating at 40 kV and 35 mA at room temperature. Conventional reference–intensity ratio (RIR) method [53] was used for the semi-quantitative analysis. The degree of crystallinity of the mortar samples was calculated based on the ratio between the area of diffraction peaks over the total area of the diffraction peaks and amorphous halos.

##### Scanning Electron Microscopy (SEM) and Energy Dispersive X-ray Spectroscopy (EDX) Measurements

SEM-EDX analysis was performed at room temperature using a scanning electron microscope (VEGAS 3 SBU, Tescan, Brno-Kohoutovice, Czech Republic) with a Quantax EDX XFlash (Bruker, Karlsruhe, Germany) detector. Samples of ~4 mm^2^ were mounted with carbon tape on an SEM stub.

#### 2.2.3. NMR Relaxometry Measurements of the Prepared Mortars

From intact prismatic samples prepared for standard mortar and mortar with 50% GGBS, a cylindrical sample with a length of 20 mm and a diameter of 9.5 mm was extracted to determine the relative pore size distribution with the NMR relaxometry technique [32]. After drying in the oven, the samples were placed in 10 mm diameter glass tubes and sealed. The first NMR measurement was performed to highlight the intra-C-S-H pores followed by the second measurement on cyclohexane-saturated samples to highlight the inter-C-S-H pores and the capillary pores [33]. NMR measurements were performed at 90 days of age with a low-field instrument (Minispec MQ20, Bruker, Karlsruhe, Germany) using the CPMG technique [32,33]. Before each measurement, the samples were brought into thermal equilibrium with a temperature of 35 °C. Relaxation time distributions T_2_ were extracted from the CPMG series using a numerical Laplace inversion [54,55].

#### 2.2.4. Road Concrete Mixes with Incorporated Slag (GGBS and ACBFS)

**Figure 2:**
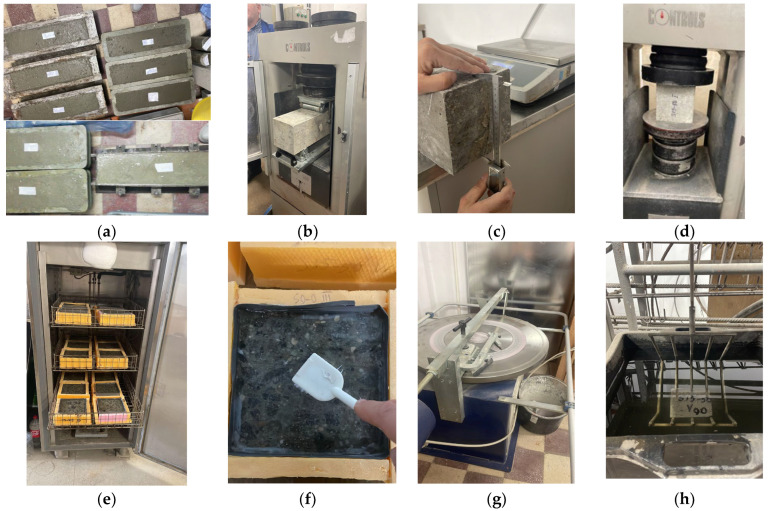
(**a**) Concrete poured in 150 × 150 × 600 mm prisms; (**b**) flexural strength test; (**c**) measuring and weighing cubes of 150 mm side; (**d**) compressive strength test; (**e**) 150 × 150 × 50 mm strips tested at 56 freeze–thaw cycles; (**f**) collecting the exfoliated mass from the surface of the sample; (**g**) Böhme abrasive wheel wear resistance test; (**h**) hydrostatic weighing of the 70 mm side cube.

A total of nine prisms measuring 150 × 150 × 600 mm were cast and left in air for 24 h. They were then preserved in water at a temperature of (20 ± 2) °C until they were 90 days old. According to SR EN 12390-5 [56], flexural strength was tested on three prismatic specimens for each compound, and the average value was formed (Figure 2a,b). The remaining samples from the flexural strength testing were used for further tests in this experimental study. From this, cubes with a side length of 150 mm, cubes with a side length of 70 mm, and strips with a thickness of 50 mm were cut.

The compressive strengths according to SR EN 12390-3 [57] and the loss of compressive strength between the control samples preserved in water and the samples tested with 100 freeze–thaw cycles were determined on cubes with a side length of 150 mm according to SR 3518 [58], (Figure 2c,d).

Mass loss due to exfoliating in the presence of de-icing agents (3% NaCl) after 56 freeze–thaw cycles according to SR CEN/TS 12390-9 [59] was determined on strips with a cross-section of 150 × 150 mm and a thickness of 50 mm (Figure 2e,f). On the samples tested with 56 freeze–thaw cycles, the migration coefficient of the sodium chloride solution was determined using the colorimetric method according to the NT BUILD 492 test [60].

The volume loss from wear according to SR EN 1338 [61] (Figure 2g) and the permeable pore content according to ASTM C 642 [62] were determined on cubes with a side length of 71 mm (Figure 2h).

The freshly split fragments were sprayed with 1% phenolphthalein solution to determine the carbonation corrosion resistance in accordance with SR CR 12793 [63].

The quality of the concrete was assessed according to the requirements indicated in Table 6 for freeze–thaw resistance and the conditions indicated in Table 7 for wear resistance.

**Figure 3:**
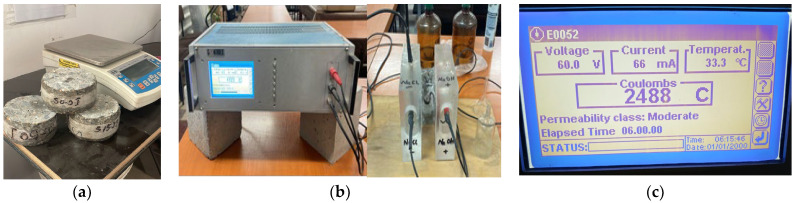
(**a**) Specimens protected with epoxy material on the side surface; (**b**) image during RCPT test; (**c**) the electric charge passed through the sample S 0/0 after 6 h.

Chloride corrosion resistance was evaluated on cylindrical specimens 100 mm in diameter and 50 mm thick using the RPCT test according to ASTM C 1202 [67] (Figure 3). At the end of the RCPT test, the samples were split axially and sprayed with silver nitrate (AgNO_3_), a 0.1 mol/dm^3^ (0.1 M) solution, to determine the penetration depth of chlorine (*Xd*) according to the NT BUILD 492 test [60]. Chlorine ion migration coefficients (*D*) were then calculated with the Nernst–Planck Equation (2) [60,68]:

(2) $$D=\frac{0.0239\left(273+T\right)L}{\left(V-2\right)t}(x_{d}-0.0238\sqrt{\frac{\left(273+T\right)Lx_{d}}{V-2}})$$

were:

*D*—Migration coefficient in non-equilibrium state (×10^−12^ m^2^/s);
*V*—Applied voltage (V);
*T*—Mean value between initial and final temperature in the anolytic solution, (°C);
*L*—Sample thickness (mm);
*Xd*—Average chlorine penetration depth (mm).

The correlation between the past electrical charge Q and the penetrability of chlorine ions is presented in Table 8:

## 3. Results and Discussion

### 3.1. Mortar Mixes with GGBS

#### 3.1.1. The Influence of HAI of GGBS on the Composition of Mortars

**Figure 4:**
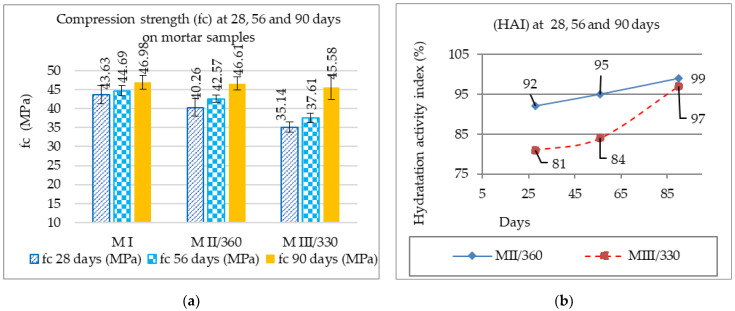
(**a**) The compressive strengths of mortars; (**b**) evolution (HAI) as a function of age from 28 to 90 days.

The compressive strengths of the standard mortar MI and the test mortars MII/360 and MIII/330 are shown in Figure 4a. The values determined for the tests at 28 days of age on cement mortars were taken from the previous work [69].

As can be seen, the highest increase in the compressive strengths from 28 to 90 days was 22.90% for the MIII/330 mortar and 13.62% for MII/360, respectively, while the increase for MI was 7.13%. In Figure 4b, it can be seen that the HAI of the 50% GGBS mortar samples increased with age [24]. The slower hydration reaction and lower heat release rate of the GGBS compared to Portland cement [70] resulted in a low HAI at 28 days of age. But at 90 days of age, the HAI for the slag mortar reached a value close to a strength class of 100 according to ASTM C989 [71], exceeding the value at 28 days (strength class 80). The higher specific surface area of the MII/360 mortar had a significant impact on increasing the reaction capacity and hydration rate [24,72,73] at the reference ages of 28 and 56 days but had a slightly smaller impact at the 90-day age. The value of the HAI of 97 for the MIII/330 mortar and close to the value of 99 for MII/360 confirms the increase in the hydration rate at 90 days.

For quality control in the production of mortar/concrete samples, the standard deviation has been plotted in Figure 4a to show how much the individual values vary compared to the mean compressive strength value. In addition, the values of the CoV, the coefficient of variation (%), in Table 9, calculated by dividing the standard deviation by the mean, show that most of the results are below the 10% limit [74,75] with the exception of MII/360 at 28 days and MIII/330 at 90 days, which are below 15% as in the papers [74,76].

#### 3.1.2. X-ray Diffraction

**Figure 5:**
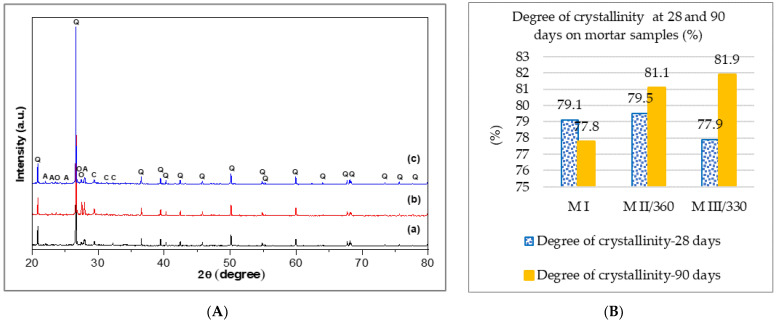
(**A**) XRD patterns of (a) M I, (b) M II/360, and (c) M III/330 mortar samples at 90 days. Note: Q—quartz, A—albite, O—orthoclase, and C—calcium silicate; (**B**) degree of crystallinity at 28 and 90 days on mortar samples (%).

Figure 5A shows the XRD patterns of the mortar samples at the age of 90 days. The XRD patterns of the mortar samples at 90 days presented in Figure 5A indicate the presence of quartz (SiO_2_) as the main crystalline phase, accompanied by albite (NaAlSi_3_O_8_), orthoclase K(AlSi_3_O_3_), and traces of calcium silicate hydrate (CaH_2_O_4_Si). At the age of 90 days, the portlandite and ettringite, which are characteristic of early cement hydration, were not remarked [77].

Figure 5B shows the evolution of the degree of crystallinity for the investigated samples up to the age of 90 days and those previously reported at 28 days [69], the difference up to 100% representing the amorphous fraction [78].

The results indicate that replacing cement with 50% GGBS increases the degree of crystallinity from 28 to 90 days, while the degree of crystallinity decreases for the standard mortar, on the degree of reaction of the binder [79]. For the MII/360 slag mortar with a higher specific surface at the age of 28 days, it approaches that of the standard mortar. In MIII/330 slag mortar, the degree of reaction has a high growth rate from 28 to 90 days above that of MII/360 mortar and standard mortar.

The decrease in the degree of crystallinity from 28 to 90 days in the MI standard sample indicates a slowing down of the reaction rate of the binder and thus implicitly a slower development of the resistance structure. However, in the slag samples, the continuation of these reactions allows the resistance structure to develop after a longer curing time [80].

The concentration of defects in the crystal structures and the relative solubility of the different minerals also influence the development of the mechanical strength [81].

The XRD results correlate with the rate of increase in the mechanical strengths of the mortars, which is lower for the standard mortar and higher for the slag mortars over the period from 28 to 90 days. The higher increase in compressive strength at 90 days in the slag mortar is supported by an additional cementitious and pozzolanic reaction at a later age, which is influenced by the GGBS [82,83,84].

#### 3.1.3. Scanning Electron Microscopy (SEM) and Energy Dispersive X-ray Spectroscopy (EDX) Measurements

**Figure 6:**
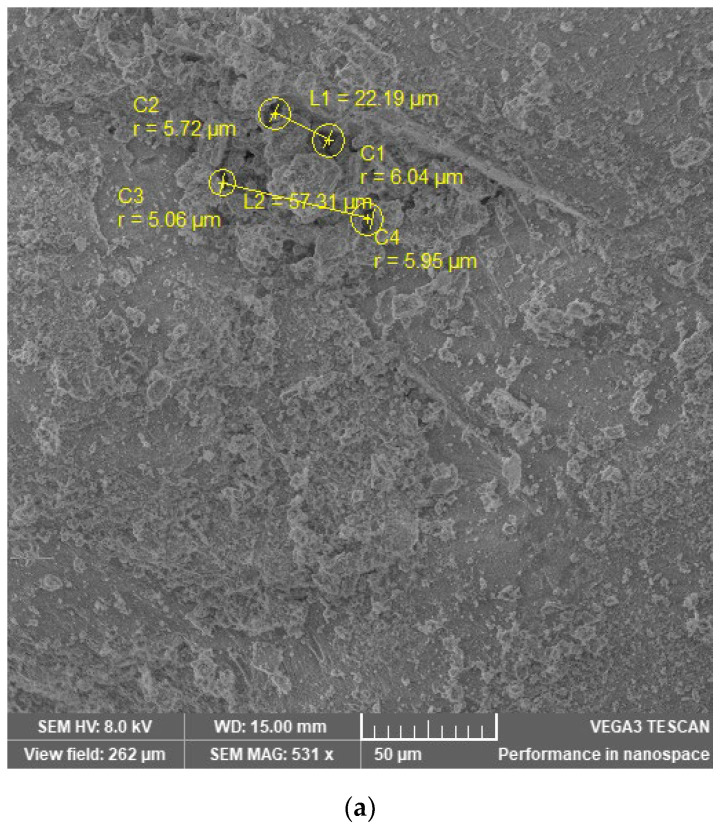

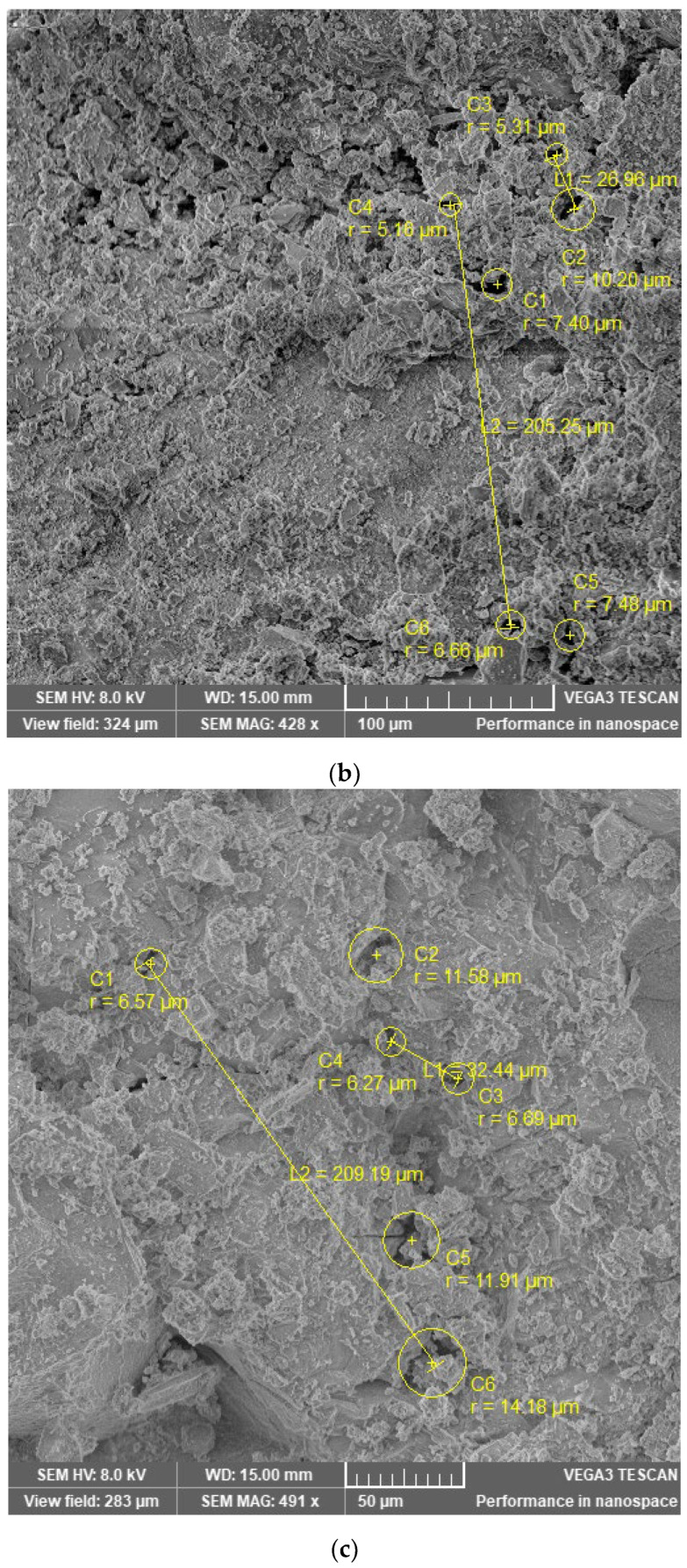
SEM images of (**a**) M I, (**b**) M II/360, and (**c**) M III/330 mortar samples at 90 days.

On the mortar samples, the SEM technique was used to characterize the structure and elemental composition on the surface of the examined samples. The images of the mortar samples M I, M II/360, and M III/330 aged 90 days (Figure 6) show that their surfaces are irregular and inhomogeneous with pore-like defects. Since the SEM technique is better suited for the study of macropores (pores > 50 nm) [85] and is limited due to the properties of the material studied, the defects observed were in the category of air voids (>several μm) [86,87,88]. Some authors use methods such as mercury intrusion porosimetry (MIP) and fractal dimensions [89,90,91] for a more complex characterization of the pore structure, but the SEM technique was used for this work, as it provides additional information about the surface structure.

Table 10 shows the measured pore size and the distances between the pores of the examined samples aged 90 days.

Larger air voids at smaller spacings indicate a more porous surface, and conversely, small pore sizes at larger spacings indicate a more compact surface [92].

At 90 days, the M I standard mortar sample (Figure 6a) shows a surface with air voids (with radii ranging from 5.06 to 6.04 µm) spaced at intervals (from 22.19 to 57.31 µm). The slag samples M II/360 (Figure 6b) and MII/330 (Figure 6c) show slightly larger air voids ((radius from 5.16 to 10.20 µm) or (from 6.27 to 14.18 µm)) compared to the standard mortar. However, the air voids are at larger spacings in the MII/360 sample (from 26.96 to 205.25 µm) and intervals in the M III/330 slag sample (from 32.44 to 209.19 µm) compared to the standard sample. This behavior may be due to the modification of the microstructure of the cement paste due to the ability of GGBS to fill the vacant pores and effectively reduce the pore volume of the matrix [84].

**Figure 7:**
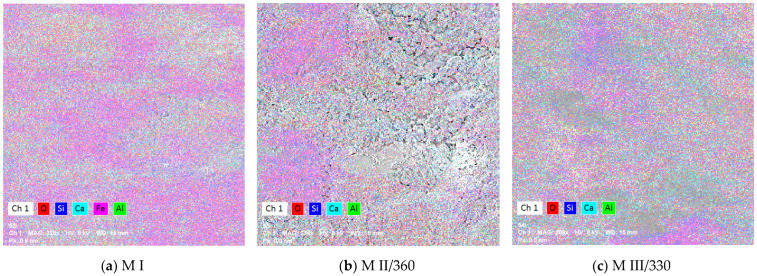
Surface mapping of (**a**) M I, (**b**) M II/360, (**c**) M III/330 mortar samples at 90 days.

**Figure 8:**
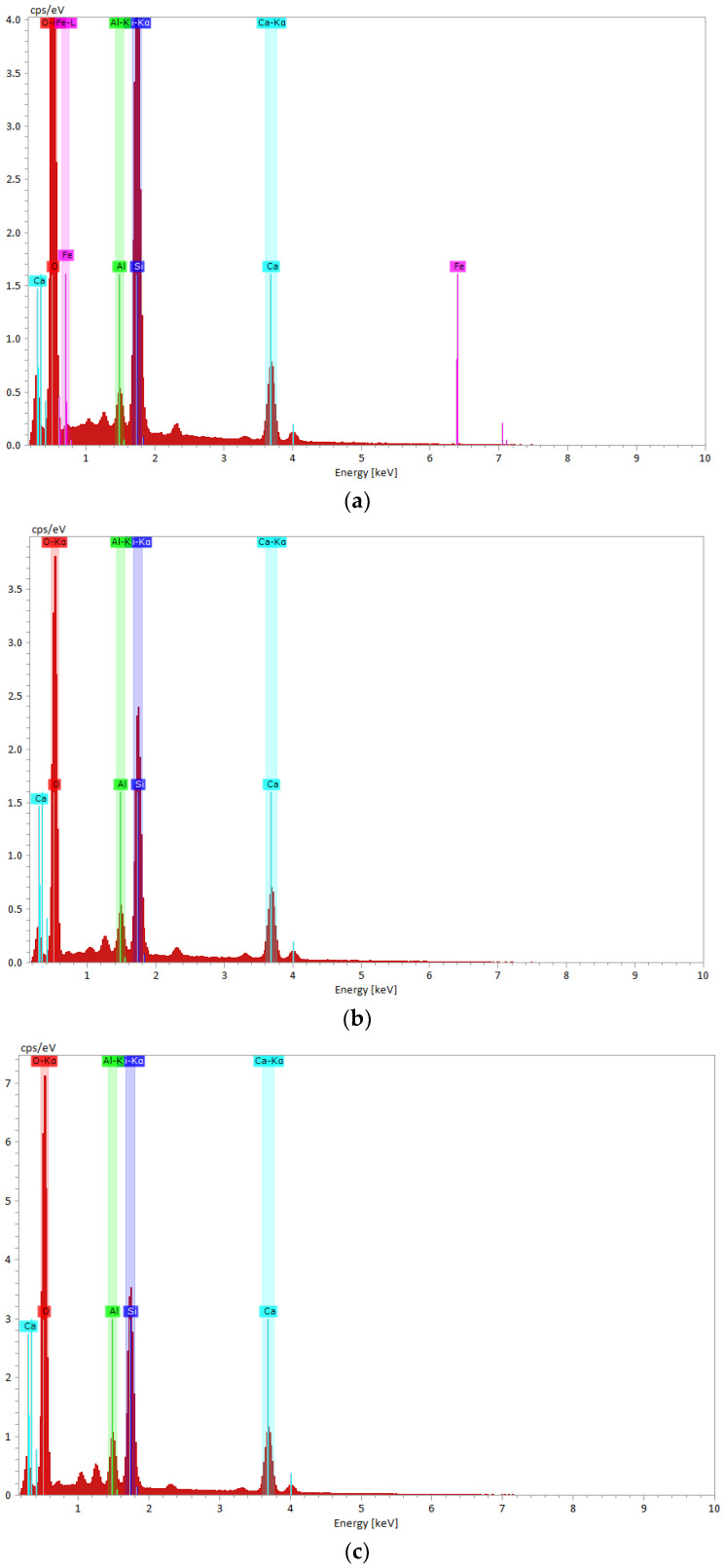
EDX spectrum of (**a**) M I, (**b**) M II/360, (**c**) M III/330 mortar samples at 90 days.

Energy-dispersive EDX spectroscopy was used to map the surfaces and identify the elemental composition of the samples previously studied via electron microscopy. The resulting images at 90 days are shown in Figure 7 and Figure 8.

**Figure 9:**
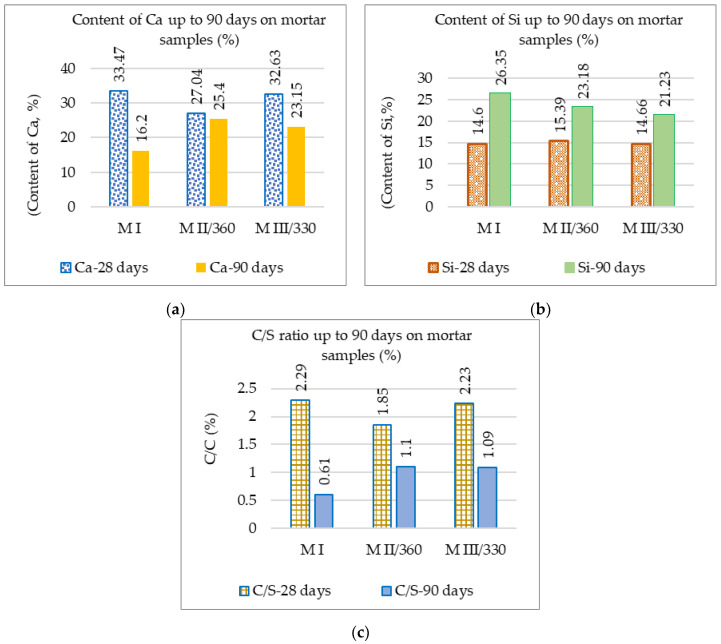
(**a**) Ca content; (**b**) Si content; (**c**) Ca/Si ratio for mortar samples M I, M II/360, and M III/330 up to 90 days.

For the three samples analyzed, the predominant elements at 28- and 90-days-old are Si and Ca. Aluminum was identified in the slag samples MII/360 and M III/330 due to the higher amounts of aluminum present in the GGBS compared to the Portland cement [93,94]. The development of the two main elements (Ca and Si) and their ratio from 28 to the 90 days are shown in Figure 9. The values recorded at 28 days of age were taken from a previous study [69].

In Figure 9a, a high concentration of Ca, the predominant phase for Portland cement, is observed in the MI standard mortar at 28 days [95]. The formation of calcium-rich gels in the standard MI mortar resulted in increased strength at 28 days compared to the slag mortars [96]. The Si content of the slag mortars is close to that of the standard mortar at 28 days and decreases slightly at 90 days (Figure 9b). At 90 days, more pronounced differences in the C/S ratio are observed in Figure 9c, which differentiates the hydrated calcium silicate (C-S-H) into different phases. The standard mortar with C/S < 1 frames the C-S-H hydrate in phase (α) with a [Ca2+] ion concentration less than 2 mmol/L, while for the slag mortars with 1 < C/S < 1.5, it frames the C-S-H in phase (β) with a [Ca2+] concentration that is between 2 and 22 mmol/L [96].

The morphology of the hydrated calcium silicate changes from weakly crystalline fibers to a reticular network [97]. The presence of the C-S-H compound in the slag mortars over a long period of time contributed significantly to increasing the compressive strengths and almost reaching the strength of the standard mortar [96,98,99] at 90 days. This behavior can be attributed to the formation of additional C-S-H gel due to the hydraulic and pozzolanic reactivity of the GGBS [84].

The EDX results correlate with those of the XRD and show the continuation of the hydration–hydrolysis reactions and the development of a strength structure after longer curing times for the slag mortar.

#### 3.1.4. NMR Relaxometry Measurements

**Figure 10:**
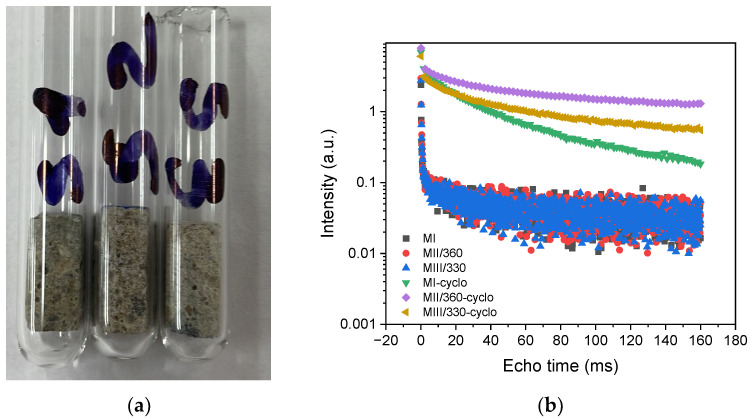
(**a**) The samples on which the NMR relaxation measurements were performed; (**b**) series of CPMG echo trains for bare and cyclohexane-saturated mortar samples M I, M II/360, M III/330 at 90 days.

The development of the CPMG echo series in the case of the bare mortar samples and cyclohexane-saturated samples at 90 days is shown in Figure 10.

**Figure 11:**
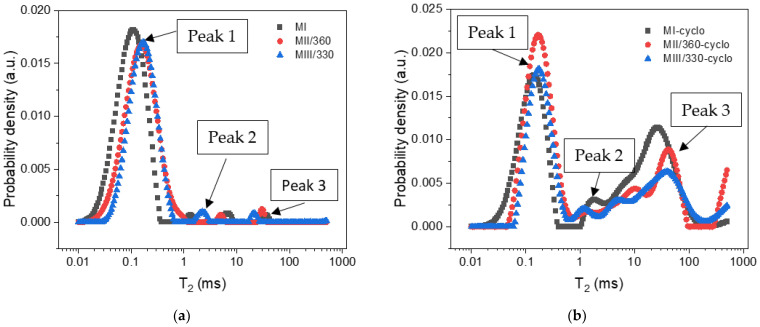
(**a**) Distribution of T_2_ relaxation times in blank samples; (**b**) distribution of T_2_ relaxation times in cyclohexane-saturated samples at 90 days.

A clearer interpretation of these results comes from the numerical Laplace transformation of the CPMG echo trains to reveal the distribution of the relaxation times [54,55]. Figure 11 shows the distributions of the relaxation times in the empty mortar samples after oven drying and the samples saturated with cyclohexane, which were determined using the numerical Laplace inversion technique.

The values for the transverse relaxation time T_2_ plotted on the horizontal axis are proportional to the relative pore size, and the values plotted on the vertical axis represent the probability density for a specific relaxation time and are expressed in arbitrary units (a.u.). The probability density indicates the probability with which a given relaxation time is present in the investigated sample. In Figure 11, three peaks can be distinguished, the position of which can be assigned to the three pore types: intra-C-S-H gel pores (Peak 1) with sizes up to 2 nm, inter-C-S-H gel pores (Peak 2) with sizes up to 10 nm, and capillary pores (Peak 3) with sizes between 50 nm ÷ 1 µm [33]. The peak area is proportional to the amount of water or cyclohexane absorbed by the pores.

It can be seen in Figure 11a that in slag samples MII/360 and MII/330, the area of Peak 1 is smaller than in control sample MI, but the pore size distribution curve is slightly shifted to higher values for T_2_. In the cyclohexane-saturated samples, in Figure 11b, in the MII/360 slag mortar, the area of Peak 1 is identified as having a higher intensity and translates to higher values than in the control sample, while in the MIII/330 sample, the area of Peak 1 is similar to the control sample. It is known that intra-C-S-H gel pores (Peak 1) with dimensions ˂ 2 nm do not cause damage to the structure of the cement paste because they are characterized by a low permeability to liquids and gases [100,101]. The inter-C-S-H gel pores in Figure 11b, corresponding to Peak 2, have a smaller surface area for the samples with slag and a distribution with lower values of T_2_ compared to the standard sample.

The area of Peak 3 corresponding to the capillary pores for the MIII/330 and MII/360 slag samples in Figure 11b is smaller than the area of the standard MI mortar, while the distribution of the T_2_ relaxation times is similar. The NMR measurements show a lower capillary porosity in the slag samples compared to the standard sample. Fewer capillary pores reduce the damage to the cement stone structure caused by the freeze–thaw phenomenon [101,102].

### 3.2. Road Concrete Mixes with Incorporated Slag (GGBS and ACBFS) Tested at 90 Days

#### 3.2.1. Mechanical Resistances, Mechanical Wear, Freeze–Thaw, and Permeable Pore Content

**Figure 12:**
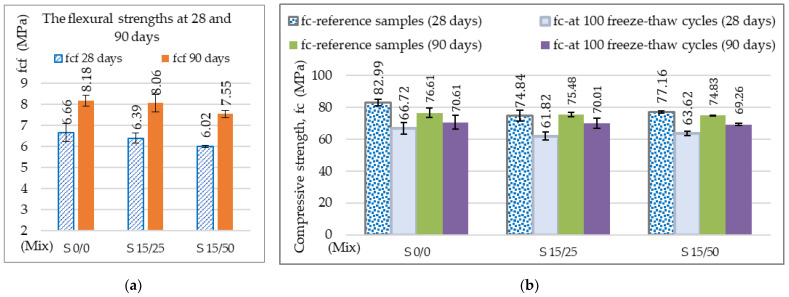

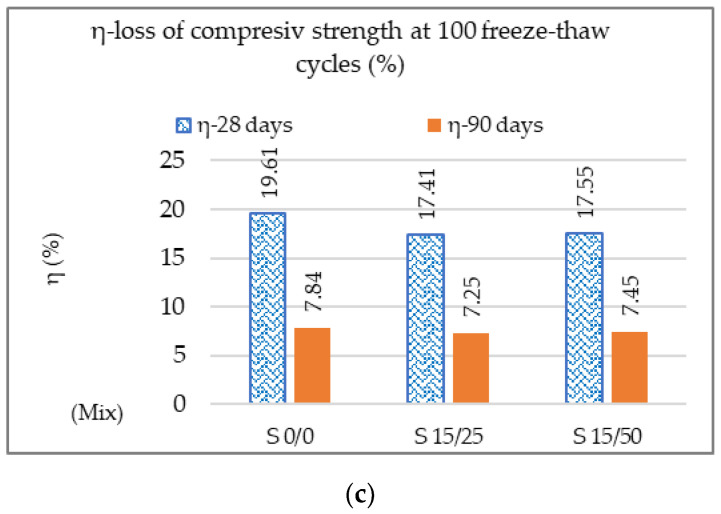
(**a**) The flexural strengths at 28 and 90 days; (**b**) the compressive strength of the reference samples and the compressive strength at 100 freeze–thaw cycles, (**c**) loss of compressive strength at 100 freeze–thaw cycles.

The evolution of the flexural strengths at 90 days, the compressive strengths of the control kept in the thermostatic chamber, and the loss of compressive strength after 100 freeze–thaw cycles are shown in Figure 12. The flexural strengths at the reference age of 28 days of the slag concrete S 15/25 and S 15/50 were 5.0 MPa, corresponding to the requirements for the very heavy traffic class BcR 5.0 [51] and the same traffic class as the reference concrete [69].

An increase in flexural strength from 28 to 90 days by 20.72% or 20.26% can be observed for the slag concrete and by 18.58% for the reference concrete (Figure 12a). In the case of the slag concrete, the flexural strength is only 1.47% lower for S15/25 and 7.7% for S15/50 compared to the reference concrete. Increasing the dosage to 50% ACBFS led to a decrease in flexural strength [97], which is due to the higher absorption and porosity c natural sand and implicitly to the increase in water demand to maintain a desired workability [19,103]. 

In Figure 12b, the compressive strength value at 90 days for the samples with slag compared of the reference composition is the lower by 1.48 (S15/25) and 2.32% (S15/50). The loss of compressive strength after 100 freeze–thaw cycles for the slag concretes is similar to that of the S0/0 reference concrete, see Figure 12c. All values obtained at 90 days of age are below the limit of 25% specified in SR 3518 [58].

**Figure 13:**
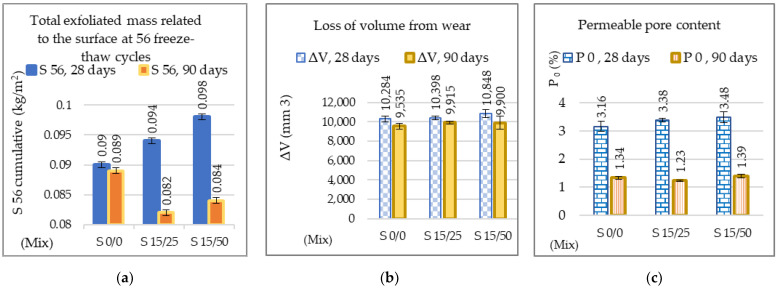
(**a**) Total exfoliated mass after 56 freeze–thaw cycles; (**b**) volume loss after mechanical wear test; (**c**) permeable pore content.

The total exfoliated mass S at 56 freeze–thaw cycles, the wear volume loss, and the permeable pore content tested at 90 days of age showed higher performance values than at 28 days, Figure 13. 

The cumulative value of the exfoliated mass of the slag samples is lower than that of the reference sample (Figure 13a); S56 is ˂0.1 kg/m^2^, which corresponds to a very good resistance to exfoliation according to SS 13 72 44 [64,65]. The loss of wear volume showed up to 3.98% higher values for the composite material S 15/25 compared to the reference composite material S 0/0 but falls within the same wear resistance class (class 4, marking I, with values ≤18,000 mm^3^/5000 mm^2^) [61] (Figure 13b). The permeable pore content recorded low values between 1.39% and 1.23% at 90 days, which corresponds to compact concretes. Slag concrete S15/25 has an 8.21% lower content of permeable pores than the reference concrete S0/0 (Figure 13c). A partial replacement of natural sand with a lower proportion of ACBFS (25%) could lead to an optimal balance between the packing density and void content in the composite, thus creating a denser microstructure [23,104]. The reduction in the performance parameters of concrete S15/50 compared to concrete S15/25 can be explained by the increase in porosity. At 90 days of age, the increase in porosity in the S 15/50 composite was not affected by the slower hydration of GGBS [105]. The reason for this was the increase in the percentage of the substitution of natural sand by the ACBFS aggregate, which has a higher water absorption coefficient than natural sand.

**Figure 14:**
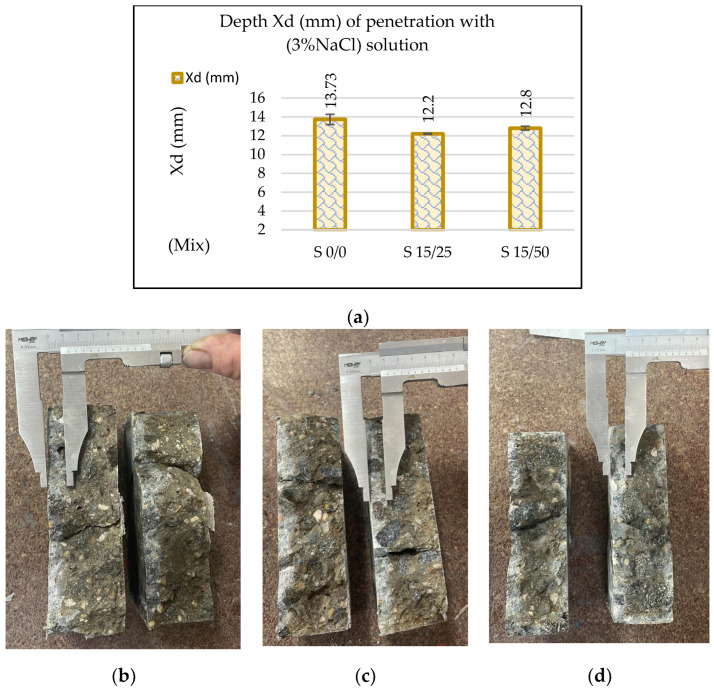
(**a**) Penetration depth of (3%NaCl) solution in samples tested with 56 freeze–thaw cycles; images of Xd measurement (**b**) for sample S 0/0; (**c**) for sample S 15/25; and (**d**) for sample S 15/50.

Figure 14 shows the penetration depth (Xd) of the sodium chloride solution used as a thawing agent in a test of 56 freeze–thaw cycles. The measurement was made after splitting and spraying the concrete strips with a size of 150 × 150 × 50 mm with silver nitrate (AgNO_3_). Compared to the reference concrete S0/0, a decrease of 11.14% was observed for sample S15/25 and a decrease of 6.77% for sample S15/50, which indicates a better resistance of the slag concretes to repeated freeze–thaw cycles with deicing agents.

#### 3.2.2. Resistance to Corrosion from Chlorides and from Carbonation at 90 Days

**Figure 15:**
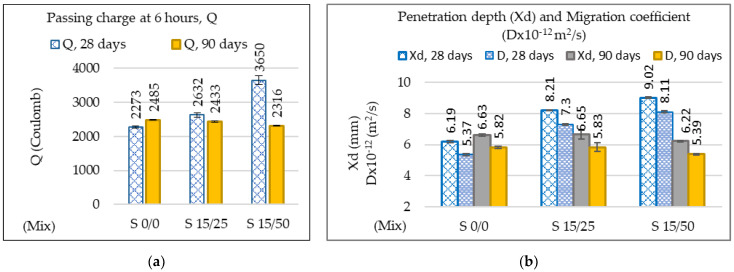
(**a**) Electric charge Q recorded at 6 h; (**b**) penetration depth Xd and migration coefficient Dx (10^−12^ m^2^/s) of chlorine ions.

The average values of the electrical pass-through charges Q resulting from the rapid chlorine penetration test (RCPT) and the average penetration depth of the chlorine ions measured on three samples are shown in Figure 15. The pass-through electrical charges recorded values between (2316 ÷ 2485) Coulombs for all the prepared mixes (Figure 15a), falling within the moderate class of chloride ion permeation concretes according to ASTM C 1202 [67].

**Figure 16:**
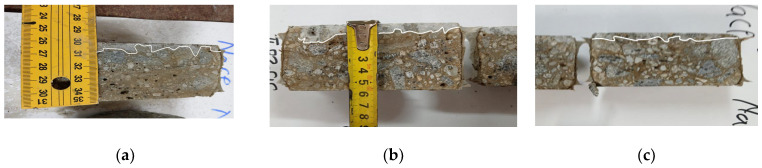
Chlorine ion migration front at the age of 90 days **(a)** for sample S 0/0; (**b**) for sample S 15/25; and (**c**) for sample S 15/50.

At the age of 90 days, the passing electrical charges Q decrease by up to 6.8%, the penetration depth of chlorine ions Xd by up to 6.18%, and the migration coefficient Dx 10^−12^ (m^2^/s) by up to 7.39% (S15/50) compared to the reference concrete (Figure 15b and Figure 16). The results obtained show that the concretes with incorporated GGBS have an increased resistance to the penetration of chlorine ions compared to conventional concretes, which is similar to the investigations [37,106].

#### 3.2.3. Carbonation Corrosion Resistance at 90 Days

**Figure 17:**
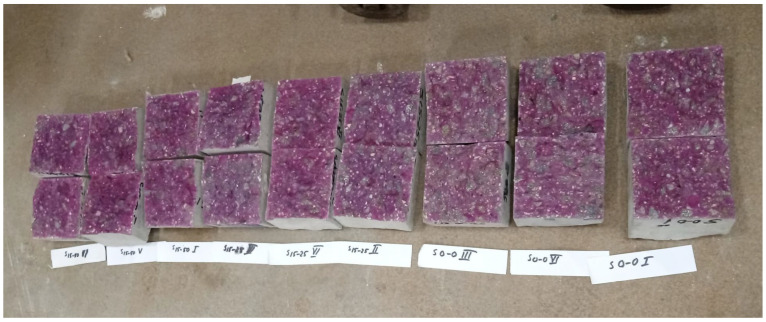
Carbonation progress after one hour of spraying the phenolphthalein solution.

The image in Figure 17 shows the samples after one hour of spraying the phenolphthalein solution. The color of the indicator solution (red-purple) on the contour areas of the surfaces remained identical to the interior area of the tested samples. The diffusion of carbon dioxide from the cement matrix was hampered by the increased compactness of the cement paste in the concrete [107,108]. The image below shows that the samples tested were not affected by carbonation corrosion.

The results obtained as the average of the three samples tested for each mixture and the standard deviation are shown in Figure 12, Figure 13, Figure 14 and Figure 15. The CoV (%), coefficient of variation, shown in Table 11 is below 10% for most of the results with the exception of two results that are below the accepted limit of 15% according to Appendix B of SR EN 206 [52].

## 4. Conclusions

The purpose of this paper is to show the potential role of GGBS and ACBFS in the composition of pavement concretes after a longer curing period. The optimal proportions of GGBS as an SCM and ACBFS aggregate as a substitute for natural sand in the paving concrete composition were determined with the performance at 90 days being similar to that of the reference concrete. To obtain a clearer picture of the influence of the GGBS on the concrete composite, mortars containing 50% GGBS were prepared, and the behavior was highlighted by evaluating the HAI and the microstructure of the composite using SEM, XRD, EDX, and NMR. The performance of the road slag concrete was evaluated using two mixes in which GGBS replaced the cement in the same percentages of 15%, and ACBFS aggregates replaced 25% and 50% of natural sand.

In summary, the results of the tests carried out on mortar and concrete show the following:

The GGBSs with specific surface areas of 360 m^2^/kg and 330 m^2^/kg were classified according to the HAI at strength class 80 at 28 days and strength class 100 at 90 days according to ASTM C989.

The degree of crystallinity determined via X-ray diffraction correlates with the rate of increase in the mechanical strength of the mortars and is lower for the standard mortar and higher for the slag mortars at 90 days.

The air voids identified via the SEM measurements in the slag mortars are larger than in the standard MI mortar but are more widely spaced, suggesting a more compact surface.

The presence of different C-S-H forms in the slag mortars, identified via EDX measurements at 90 days, justifies the increase in mechanical strength to values similar to those of the standard mortar.

The NMR measurements show a lower content of capillary voids compared to the standard mortar, resulting in a better resistance to repeated freeze–thaw cycles.

The durability parameters (strength losses and the amount of exfoliation after the freeze–thaw cycles, as well as their resistance to corrosion by chlorine ion penetration and by carbonation) at 90 days of the two composites S15/25 and S15/50 were at the same level or higher than the reference concrete value.

The S15/25 composite achieved similar mechanical strengths and volume loss from wear as the reference concrete, but the values were lower for the S15/50 concrete.

This study confirms optimum proportions for the S15/25 composite when the Portland cement replacement is 15% GGBS and natural sand is 25% ACBFS. In addition, the use of GGBS with a specific surface area equal to that of Portland cement results in environmentally friendly road concrete with low production costs.

The hydration reactions of GGBS as a cement substitute continuate over a longer curing period, so the reference age of 90 days is the appropriate option. This experimental study responds to the desires of the circular economy, and the results can be successfully used to obtain ecological and economical road concretes.

## Figures and Tables

**Table 1:** Oxidative analysis of GGBS and ACBFS (%).

Element	SiO_2_	Al_2_O_3_	MnO	MgO	CaO	Fe_2_O_3_	FeO	Na_2_O	K_2_O	Other
GGBS (%)	38.10	9.50	0.23	8.10	40.80	0.56		0.30	0.68	1.73
ACBFS (%)	39.01	9.14	0.51	7.57	41.37		0.58	0.01	0.64	1.17

**Table 2:** The composition of mortars.

Materials	M I	M II/360	M III/330
Binder-specific surface area	331 m^2^/kg	360 m^2^/kg	330 m^2^/kg
Portland cement, (g)	450 ± 2	225 ± 1	225 ± 1
(GGBS ˂ 63 µm), (g)	-	225 ± 1	225 ± 1
(NA_0/4 mm), (g)	1350 ± 5	1350 ± 5	1350 ± 5
Water, (mL)	225	225	225

**Table 3:** Concrete composition kg/m^3^.

Material (kg/m^3^)	S 0/0 Reference Concrete	S 15/25 (15%GGBS_25%ACBFS)	S 15/50 (15%GGBS_50%ACBFS)
Cement (C)	370	314.50	314.50
(GGBS ˂ 63 µm)	-	55.50	55.50
Binder (b)	370	370	370
Water (w)	151.68	154.51	155.92
w/b	0.41	0.418	0.421
(ACBFS_0/4 mm)	-	163.44	326.90
Natural sand (AN_0/4 mm)	654.70	490.33	326.90
Coarse aggregate (AC_4/25 mm)	1215.88	1214.16	1214.18
Aggregate total	1870.58	1867.94	1867.97
Admixtures (SP-SKY 527)	5.55	6.18	6.29
Admixtures (MA 9060)	0.74	0.74	0.81

**Table 4:** Design parameters of road concrete composites.

Parameters Design	Min. Dosage Cement	(w/L)	Compaction (%)	Density (kg/m^2^)	Occluded Air (%)	fcm 28 Days MPa	fcf, fl 28 Days MPa
NE 014 and SR EN 206	360 kg/m^3^	max. 0.45	1.15 ÷ 1.35	2390 ± 30	5.0 ÷ 6.5	min. 50	min. 5.5
Results	370 kg/m^3^	0.41 ÷ 0.42	1.26 ÷ 1.33	2392 ÷ 2394	5.1 ÷ 6.4	-	-

**Table 5:** The minimum value of the HAI of the GGBS at 28 days.

SR EN 15167:1	ASTM C989 (Clasa 80)	ASTM C989 (Clasa 100)	ASTM C989 (Clasa 120)
70	75	95	115

**Table 6:** Acceptance criteria for frost resistance of concrete SS 13 72 44 [64,65], NE012-1 [66], and SR 3518 [58].

Frost-Thaw Resistance	Requirement SS 13 72 44	Requirement NE 012-1	Requirement SR 3518
Very good	m_56_ < 0.10 kg/m^2^		
Hi	m_56_ < 0.20 kg/m^2^	m_56_ < 0.50 kg/m^2^	-
(Raised)	or m_56_ < 0.50 kg/m^2^ and m_56_/m_28_ < 2		
	or m_112_ < 0.50 kg/m^2^		
Acceptable (Moderate)	m_56_ < 1.00 kg/m^2^ and m_56_/m_28_ < 2 or m_112_ < 1.00 kg/m^2^	m_56_ < 1.0 kg/m^2^	-
Unacceptable (Low)	m_56_ ≥ 1.00 kg/m^2^ and m_56_/m_28_ ≥ 2 or m_112_ ≥ 1.00 kg/m^2^	m_56_ < 2.0 kg/m^2^	-
Degree of gelling G100	-	-	Loss of compressive strength (η) ˂ 25%

**Table 7:** Wear resistance classes SR EN 1338 [61].

Class	1	3	4
Marcare	F	H	I
Criteria	No measured performance	≤20,000 mm^3^/5000 mm^2^	≤18,000 mm^3^/5000 mm^2^

**Table 8:** Interpretation of results according to ASTM C 1202 [67].

Past Electrical Charge (Coulomb)	High	Moderate	Low	Very Low
Penetrability of chlorine ions	˃4000	2000–4000	1000–2000	100–1000

**Table 9:** Coefficient of variation (CoV) (%) of compressive strengths for mortars.

Mix	MI	MII/360	MIII/330
Coefficient of variation	CoV (%)	CoV (%)	CoV (%)
Compressive strength at 28 days-fc (MPa)	10	11	8
Compressive strength at 56 days-fc (MPa)	6	4	7
Compressive strength at 90 days-fc (MPa)	8	7	13

**Table 10:** Pore measurements of mortar samples M I, M II/360, and M III/330, at 90 days.

Sample	Pore Identification Code	Pore Radius (μm)	Distance Code Li(Ci–Ci + n)	Distance (μm)
**M I**	C1	6.04	L1(C1–C2)	22.19
	C2	5.72		
	C3	5.06	L2(C3–C4)	57.31
	C4	5.95		
**M II/360**	C1	7.40		
	C2	10.20	L1(C2–C3)	26.96
	C3	5.31		
	C4	5.16	L2(C4–C6)	205.25
	C5	7.48		
	C6	6.66		
**M III/330**	C1	6.57	L2(C1–C6)	209.19
	C2	11.58		
	C3	6.69	L1(C3–C4)	32.44
	C4	6.27		
	C5	11.29		
	C6	14.18		

**Table 11:** Coefficient of variation (CoV) (%) of physical–mechanical characteristics determined for concretes at 90 days.

Mix	S 0/0	S 15/25	S 15/50
Coefficient of variation	CoV (%)	CoV (%)	CoV (%)
The tensile strength, -fct, fl (MPa)	6.1	10.4	4.4
Reference compressive strength, fc (MPa)	7.0	4.0	1.0
Compressive strength at 100 freeze–thaw, fc (MPa)	12	9.0	2.0
The exfoliated mass at 56 freeze–thaw cycles (kg/m^2^)	8.2	8.8	13.3
Loss of volume from wear (mm^3^)	5.7	2.6	7.7
Permeable pore content (%)	7.3	3.3	8.9
Passing charge at 6 h (Q)	0.7	1.7	0.6
Penetration depth Xd (mm)	2.3	8.8	1.4
Migration coefficient of chlorine ions (Dx (10^−12^ m^2^/s))	2.6	9.7	1.5

## Data Availability

All the required data that support the findings are presented in this manuscript.
